# Elevated serum albumin-to-creatinine ratio as a protective factor on clinical outcomes among critically ill patients with sepsis: a retrospective study

**DOI:** 10.3389/fmed.2024.1436533

**Published:** 2024-09-19

**Authors:** Zhenkui Hu, Chao Song, Jinhui Zhang

**Affiliations:** ^1^Department of Emergency Medicine, The Affiliated Hospital, Jiangsu University, Zhenjiang, China; ^2^Department of Critical Care Medicine, The Affiliated Hospital, Jiangsu University, Zhenjiang, China

**Keywords:** sepsis, albumin, creatinine, albumin-to-creatinine ratio, mortality

## Abstract

**Background:**

The aim of this study was to examine the prognostic significance of serum albumin-to-creatinine ratio (ACR) in critically ill patients with sepsis.

**Methods:**

This retrospective study analyzed sepsis cases admitted to the Affiliated Hospital of Jiangsu University between January 2015 and November 2023. The patients were divided into four groups based on their ACR upon admission to the intensive care unit (ICU). Laboratory data were collected at the time of ICU admission, and the primary outcome measure was in-hospital all-cause mortality. Kaplan–Meier survival curves were generated to illustrate the differences in 30−/60-day mortality among the various groups. Multivariate Cox regression models and restricted cubic splines (RCS) were utilized to explore the association between ACR and all-cause mortality in sepsis patients. Subgroup analyses were conducted to examine the impact of other covariates on the relationship between ACR and all-cause mortality.

**Results:**

A total of 1,123 eligible patients were included in the study, with a median ACR of 0.169. The in-hospital mortality rate was 33.7%, the ICU mortality rate was 31.9%, and the 30-day mortality rate was 28.1%. Kaplan–Meier survival analysis demonstrated that patients with higher ACR had a significantly lower risk of 30−/60-day mortality (log-rank *p* < 0.001). Multivariable Cox proportional hazards analyses revealed that ACR was an independent predictor of in-hospital death (HR: 0.454, 95% CI 0.271–0.761, *p* = 0.003), ICU death (HR: 0.498, 95% CI 0.293–0.847, *p* = 0.010), and 30-day death (HR: 0.399, 95% CI 0.218–0.730, *p* = 0.003). For each 1-unit increase in ACR, there was a 1.203-fold decrease in the risk of death during the hospital stay. The RCS curve illustrated a non-linear negative correlation between ACR and in-hospital mortality (p for non-linear =0.018), ICU mortality (*p* for non-linear =0.005), and 30-day mortality (*p* for non-linear =0.006). Sensitivity analysis indicated consistent effect sizes and directions in different subgroups, confirming the stability of the results.

**Conclusion:**

Low ACR levels were identified as independent risk factors associated with increased in-hospital, ICU, and 30-day mortality in sepsis patients. ACR can serve as a significant predictor of the clinical outcome of sepsis.

## Introduction

Sepsis is a life-threatening condition characterized by organ dysfunction resulting from an imbalanced immune response to infection (1). In 2017, the World Health Organization (WHO) recognized sepsis as a global health priority (2), and the Global Burden of Disease (GBD) project estimated that nearly 50 million cases of sepsis and 11 million sepsis-related deaths occurred worldwide in the same year (3). It was worth noting that sepsis remained a significant reason for ICU admissions in low and middle-income countries, where mortality rates as high as 80% had been reported (4). Furthermore, sepsis survivors faced ongoing challenges, with a 15% mortality rate within the first year after discharge and 6–8% in the subsequent 5 years (5, 6). Early identification and intervention for sepsis patients at higher risk of mortality were crucial for improving prognosis (7). Therefore, early risk stratification played a vital role in helping clinicians determine appropriate treatment strategies and long-term management approaches.

Many biomarkers had been utilized in predicting the prognosis of sepsis patients, including Pentraxin (PTX-3), mid-regional pro adrenomedullin (MR-proADM), and soluble tumor necrosis factor receptor type 1 (sTNFR1) (8–10). However, these tools were either expensive or not readily accessible. It was well-known that inflammation and oxidative stress played crucial roles in the pathogenesis of sepsis (11, 12). Serum albumin (Alb), a significant plasma protein, possessed important physiological functions such as immune regulation, endothelial stability, inflammation response, and antioxidant effects (13). There was mounting evidence indicating a close association between serum Alb levels and infectious diseases like sepsis, COVID-19, and acute pancreatitis (14–16). Nevertheless, it should be noted that the patient’s nutritional status or chronic inflammation can also impact albumin levels, which may introduce limitations when relying solely on albumin levels for prediction (17). Furthermore, serum creatinine, an indicator of renal function, was linked to oxidative stress, endothelial function, and inflammation (18, 19). Elevated levels of serum creatinine upon admission could aid in predicting the onset of adverse events in sepsis patients (20). However, it was important to consider that abnormal creatinine levels may also result from chronic kidney disease. Therefore, relying solely on creatinine prediction may not guarantee reliable outcomes. Remarkably, the serum albumin-to-creatinine ratio (ACR), a novel composite ratio, had emerged as an easily obtainable parameter that had shown promising predictive utility in assessing adverse outcomes among patients with ST-elevation myocardial infarction (STEMI), heart transplantation, and diabetes (21–23). To the best of our knowledge, the effectiveness of serum ACR as a predictive marker had not been investigated in sepsis patients. Hence, the main objective of this retrospective study was to analyze the correlation between serum ACR and prognosis in critically ill sepsis patients.

## Methods

### Study design and setting

This retrospective study included adult patients (aged ≥ 18 years) who were admitted to the ICU of the Affiliated Hospital of Jiangsu University between January 2015 and November 2023. The study protocol underwent review by the ethical review board of the Affiliated Hospital of Jiangsu University (No. KY2023K1007). Written informed consent was obtained from all participating patients.

### Study population

**Figure 1:**
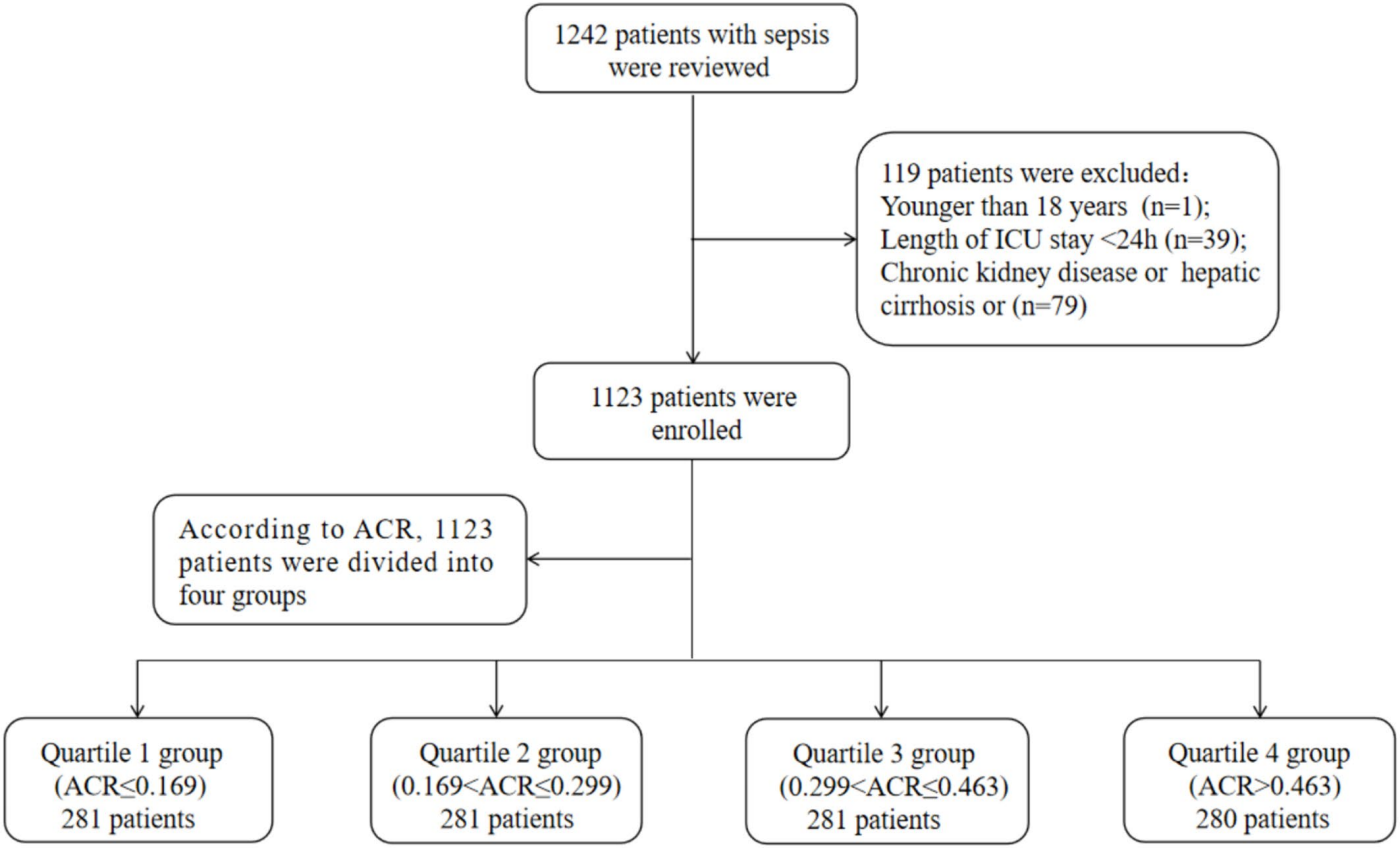
Flow of included patients through the trial. ACR, albumin-to-creatinine ratio; ICU, Intensive Care Unit.

Patients were included in this study if they met the following criteria: a diagnosis of sepsis based on the sepsis 3.0 diagnostic criteria, which included infection and a Sequential Organ Failure Assessment (SOFA) score of ≥2 (24). If a patient had multiple admission records, data from their first admission were used. Patients were excluded if they met any of the following conditions: being under 18 years old, staying in the ICU for less than 24 h, or having chronic kidney disease or hepatic cirrhosis. Ultimately, a total of 1,123 patients were included in the final analysis. The detailed inclusion and exclusion process was depicted in Figure 1.

### Data collection

The potential variables for this study were extracted from the electronic medical record system. The following basic characteristics of all patients were collected upon admission to the hospital: age, gender, body mass index (BMI), smoking history, hypertension, diabetes, coronary artery disease, chronic obstructive pulmonary disease (COPD), and cerebral infarction. The following laboratory test parameters were collected at the day of ICU admission: white blood cell (WBC), neutrophil (Neu), lymphocyte (Lym), monocyte (Mon), hemoglobin (Hb), platelet (PLT), C-reactive protein (CRP), total bilirubin (Tbil), alanine transaminase (ALT), aspartate aminotransferase (AST), albumin (Alb), glucose, creatinine, blood urea nitrogen (BUN), uric acid, D-dimer, potassium, and lactate. We selected the first set of parameters if the variables included were measured more than once on the day of ICU admission. Additionally, the Acute Physiology and Chronic Health Evaluation II (APACHE II) score and SOFA score were calculated within the first 24 h of ICU admission. The treatments received, including continuous renal replacement therapy (CRRT), vasoactive drugs, and invasive ventilation, were also recorded. The follow-up duration was defined as the period from ICU admission until death or discharge. The serum albumin-to-creatinine ratio at presentation was calculated as follows: serum albumin (g/L)/serum creatinine (μmol/L). Subsequently, the patients were categorized into four groups based on their ACR quartile range: Quartile 1 group (ACR ≤ 0.169, ≤ 25th), Quartile 2 group (0.169 < ACR ≤ 0.299, 25th-50th), Quartile 3 group (0.299 < ACR ≤ 0.463, 50th–75th), and Quartile 4 group (ACR > 0.463, > 75th).

### Primary outcome and secondary outcomes

The primary outcome of the current study was all-cause mortality occurring during the hospital stay. The secondary endpoints included mortality specifically within the ICU and mortality within 30 days following admission to the ICU.

### Statistical analysis

The statistical analysis was conducted using SPSS version 26.0, GraphPad Prism 10.0, and R software version 4.1.3. Statistical significance was defined as *p* < 0.05. Continuous variables were presented as mean ± standard deviation (mean ± SD) for normally distributed data, or median (25th and 75th) for non-normally distributed data. Categorical variables were expressed as numbers and percentages. The Mann–Whitney U test or Student’s t-test was employed to compare continuous variables, while the chi-square test was used for categorical variables. To visualize the time to the occurrence of 30−/60-day mortality, Kaplan–Meier analysis was performed, and the log-rank test was used to compare differences between the four groups based on the ACR. Cox proportional hazard models were employed to calculate the hazard ratio (HR) and 95% confidence interval (CI) to explore the association between ACR and 30-day, ICU, and in-hospital mortality. Three Cox proportional hazards regression models were constructed to evaluate the independent association of ACR with the primary endpoint. Model 1 was an unadjusted model with no variables adjusted. Model 2 was adjusted for age, gender, BMI, smoking, hypertension, and diabetes. Model 3 was adjusted for age, gender, BMI, smoking, hypertension, diabetes, WBC, Neu, Lym, CRP, ALT, AST, glucose, APACHE II score, and SOFA score. ACR was included in the models as either a continuous variable or a categorical variable. Moreover, restricted cubic spline (RCS) regression with three knots (10th, 50th, and 90th percentiles) was applied to assess the potential non-linear association between ACR and 30-day, ICU, and in-hospital mortality. Subgroup analysis was conducted based on age, gender, BMI, smoking, hypertension, diabetes, and SOFA score. *p* values for interaction were calculated to investigate the effect of each subgroup on the outcome.

## Results

### Baseline characteristics

The baseline clinical and biochemical characteristics of the 1,123 individuals were assessed in this study, with 707 (63.0%) being males (Table 1). The median levels of Alb, creatinine, and ACR were determined to be 28.2 g/L (IQR: 24.2–33.2), 92.6 μmol/L (IQR: 63.7–153.1), and 0.300 (IQR: 0.169–0.463), respectively. The in-hospital, ICU, and 30-day mortality rates were observed to be 33.7, 31.9, and 28.1%, respectively. It was noted that patients with higher ACR levels exhibited a lower proportion of males and coronary artery disease, but a higher prevalence of COPD. Furthermore, these patients demonstrated elevated levels of Lym, PLT, Hb, and Alb, along with reduced levels of WBC, Neu, CRP, Tbil, ALT, AST, glucose, creatinine, BUN, uric acid, D-dimer, potassium, and lactate. Notably, they also displayed a lower severity of illness as measured by APACHE II score and SOFA score, as well as a decreased utilization of CRRT and vasoactive drugs.

**Table 1:** Characteristics and outcomes of participants categorized by ACR.

Variables	Overall	Q1 group (ACR ≤ 0.169)	Q2 group (0.169 < ACR ≤ 0.299)	Q3 group (0.299 < ACR ≤ 0.463)	Q4 group (ACR > 0.463)	*p*-value
N	1,123	281	281	281	280	
Age, years	75 (65–84)	75 (67–83)	76 (66–85)	77 (66–86)	73 (61–83)	0.096
Male, *n* (%)	707 (63.0)	187 (66.5)	174 (61.9)	192 (68.3)	154 (55.0)	0.005
BMI, kg/m^2^	22.49 (20.08–25.21)	22.41 (20.10–24.50)	22.60 (19.96–25.64)	22.83 (20.42–25.39)	22.49 (16.70–24.89)	0.188
Smoking, *n* (%)	229 (20.4)	57 (20.3)	55 (19.6)	65 (23.1)	52 (18.6)	0.579
Comorbidities, *n* (%)						
Hypertension	579 (51.3)	152 (54.1)	153 (54.4)	146 (52.0)	128 (45.7)	0.138
Diabetes	309 (27.5)	93 (33.1)	75 (26.7)	75 (26.7)	66 (23.6)	0.080
Coronary artery disease	116 (10.3)	42 (14.9)	30 (10.7)	25 (8.9)	19 (6.8)	0.012
COPD	87 (7.7)	15 (5.3)	18 (6.4)	22 (7.8)	32 (11.4)	0.040
Cerebral infarction	161 (14.3)	37 (13.2)	43 (15.3)	35 (12.5)	46 (16.4)	0.506
Laboratory tests						
WBC *10^9^ /L	11.4 (7.4–17.1)	13.1 (7.9–20.0)	11.9 (6.8–17.1)	10.7 (7.5–16.8)	10.6 (7.4–15.0)	0.004
Neu *10^9^ /L	10.1 (6.3–15.5)	12.2 (6.8–18.5)	10.5 (6.0–15.7)	9.5 (6.3–15.5)	9.3 (6.2–13.5)	<0.001
Lym *10^9^ /L	0.6 (0.3–0.9)	0.5 (0.3–0.8)	0.5 (0.3–0.9)	0.6 (0.4–0.9)	0.6 (0.4–1.0)	<0.001
Mon *10^9^ /L	0.4 (0.2–0.7)	0.4 (0.2–0.7)	0.4 (0.2–0.7)	0.4 (0.2–0.7)	0.4 (0.2–0.6)	0.598
Hb, g/dL	115 (97–130)	109 (89–128)	112 (97–129)	117 (100–131)	118 (101–133)	0.001
PLT *10^9^ /L	149 (95–214)	114 (67–172)	146 (92–206)	160 (109–223)	186 (130–251)	<0.001
CRP, mg/L	104.2 (42.0–163.2)	144.5 (90.0–214.7)	112.0 (50.9–161.8)	91.5 (33.4–154.8)	72.1 (21.3–116.2)	<0.001
Tbil, μmol/L	17.4 (10.9–28.2)	20.2 (12.4–34.4)	18.6 (11.4–32.2)	16.0 (10.6–23.8)	15.3 (9.0–23.6)	<0.001
ALT, U/L	32.0 (21.0–56.0)	40.0 (23.0–78.0)	32.0 (22.0–59.5)	29.0 (20.0–45.0)	30.0 (20.0–51.0)	<0.001
AST, U/L	38.1 (23.9–73.0)	55.9 (29.0–146.0)	41.0 (24.0–84.5)	34.5 (22.1–59.0)	31.0 (22.0–60.0)	<0.001
Alb, g/L	28.2 (24.2–33.2)	24.6 (21.1–28.8)	27.2 (23.8–31.8)	29.5 (25.2–33.7)	32.0 (28.5–36.3)	<0.001
Glucose, mmol/L	8.2 (6.6–11.8)	9.0 (6.8–13.9)	8.3 (6.6–12.2)	8.1 (6.5–11.4)	7.7 (6.4–10.4)	0.003
Creatinine, μmol/L	92.6 (63.7–153.1)	224.0 (169.8–344.8)	119.9 (101.5–136.8)	76.1 (65.9–88.9)	52.4 (44.1–62.4)	<0.001
BUN, mmol/L	8.89 (6.04–13.95)	17.83 (12.06–24.20)	10.01 (7.67–14.43)	7.03 (5.48–9.48)	5.70 (4.30–7.70)	<0.001
Uric acid, μmol/L	286.9 (192.3–411.7)	441.3 (327.5–561.7)	311.1 (255.0–429.4)	256.0 (169.4–327.1)	189.3 (135.6–251.4)	<0.001
D-dimer, mg/L	4.2 (2.1–8.4)	6.2 (3.2–12.7)	5.6 (2.9–9.6)	3.9 (2.1–7.1)	2.6 (1.5–5.1)	<0.001
Potassium, mmol/L	3.7 (3.3–4.2)	3.9 (3.5–4.7)	3.6 (3.2–4.2)	3.7 (3.3–4.1)	3.6 (3.3–3.9)	<0.001
Lactate, mmol/L	2.1 (1.4–3.6)	2.9 (1.9–5.3)	2.4 (1.7–4.0)	1.9 (1.3–2.9)	1.7 (1.2–2.4)	<0.001
ACR	0.300 (0.169–0.463)	0.118 (0.077–0.145)	0.230 (0.202–0.268)	0.379 (0.340–0.419)	0.597 (0.517–0.724)	<0.001
Severity scoring						
APACHE II score	25 (19–30)	28 (22–33)	25 (19–30)	25 (19–29)	23 (18–28)	<0.001
SOFA score	12 (10–14)	13 (11–15)	12 (10–14)	12 (9–14)	11 (9–14)	<0.001
Treatments						
CRRT, *n* (%)	78 (6.9)	61 (21.7)	14 (5.0)	0 (0.0)	3 (1.1)	<0.001
Vasoactive drug, *n* (%)	748 (66.6)	235 (83.6)	214 (76.2)	163 (58.0)	136 (48.6)	<0.001
Invasive ventilation, *n* (%)	752 (67.0)	189 (67.3)	192 (68.3)	196 (69.8)	175 (62.5)	0.289
Endpoints						
30-day mortality, *n* (%)	316 (28.1)	123 (43.8)	76 (27.0)	67 (23.8)	50 (17.9)	<0.001
60-day mortality, *n* (%)	375 (33.4)	135 (48.0)	97 (34.5)	80 (28.5)	63 (22.5)	<0.001
Length of ICU stay, days	6 (3–12)	7 (4–13)	5 (3–11)	5 (3–11)	5 (2–13)	<0.001
Length of hospital stay, days	16 (11–25)	18 (9–26)	15 (10.25)	17 (12–25)	16 (11–26)	0.289
ICU mortality, *n* (%)	358 (31.9)	128 (45.6)	92 (32.7)	75 (26.7)	63 (22.5)	<0.001
Hospital mortality, *n* (%)	379 (33.7)	135 (48.0)	97 (34.5)	82 (29.2)	65 (23.2)	<0.001

ACR, albumin-to-creatinine ratio; BMI, body mass index; COPD, chronic obstructive pulmonary disease; WBC, white blood cell count; Neu, neutrophil; Lym, lymphocyte; Mon, monocyte; Hb, hemoglobin; PLT, platelet; CRP, C-reactive protein; Tbil, total bilirubin; ALT, alanine transaminase; AST, aspartate aminotransferase; Alb, albumin; BUN, blood urea nitroge; APACHE II, Acute Physiology and Chronic Health Evaluation II; SOFA, Sequential Organ Failure Assessment; CRRT, continuous renal replacement therapy; ICU, Intensive Care Unit.

**Figure 2:**
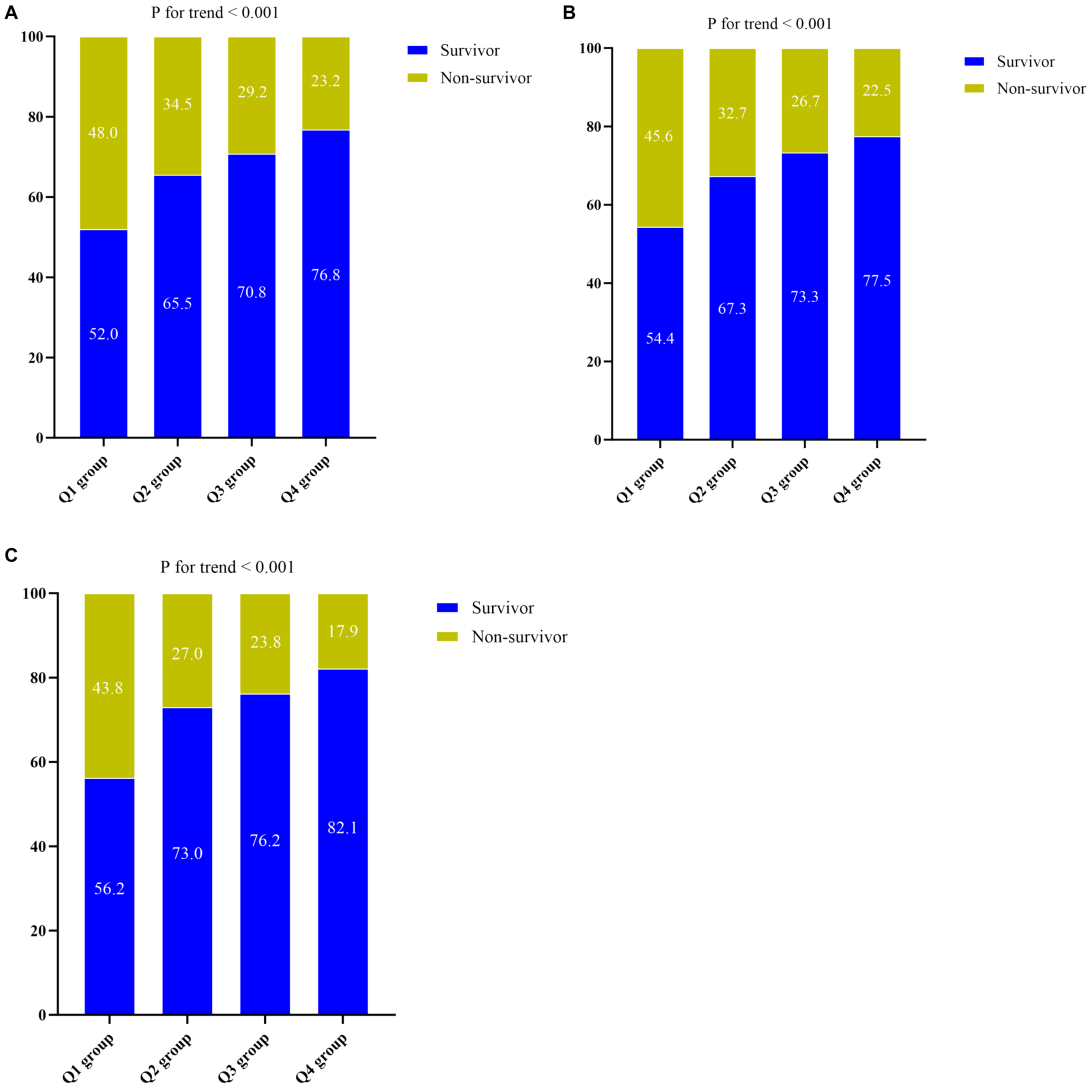
**(A)** The prevalence of hospital mortality ratio among different quartiles of ACR. **(B)** The prevalence of ICU mortality ratio among different quartiles of ACR. **(C)** The prevalence of 30-day mortality ratio among different quartiles of ACR. ACR quartiles: Q1 group (ACR ≤ 0.169); Q2 group (0.169 < ACR ≤ 0.299); Q3 group (0.299 < ACR ≤ 0.463); Q4 group (ACR > 0.463). ACR, albumin-to-creatinine ratio; ICU, Intensive Care Unit.

As ACR levels increased, there was a gradual decrease observed in the ICU length of stay (7 days vs. 5 days vs. 5 days vs. 5 days, *p* < 0.001), 30-day mortality rate (43.8% vs. 27.0% vs. 23.8% vs. 17.9%, *p* < 0.001), 60-day mortality rate (48.0% vs. 34.5% vs. 28.5% vs. 22.5%, p < 0.001), ICU mortality rate (45.6% vs. 32.7% vs. 26.7% vs. 22.5%, *p* < 0.001), and hospital mortality rate (48.0% vs. 34.5% vs. 29.2% vs. 23.2%, *p* < 0.001). Moreover, there was a notable decreasing trend in hospital mortality (*p* for trend <0.001), ICU mortality (*p* for trend <0.001), and 30-day mortality rates as ACR levels increased (*p* for trend <0.001) (Figures 2A–C).

### ACR and in-hospital, ICU, and 30-day mortality

**Figure 3:**
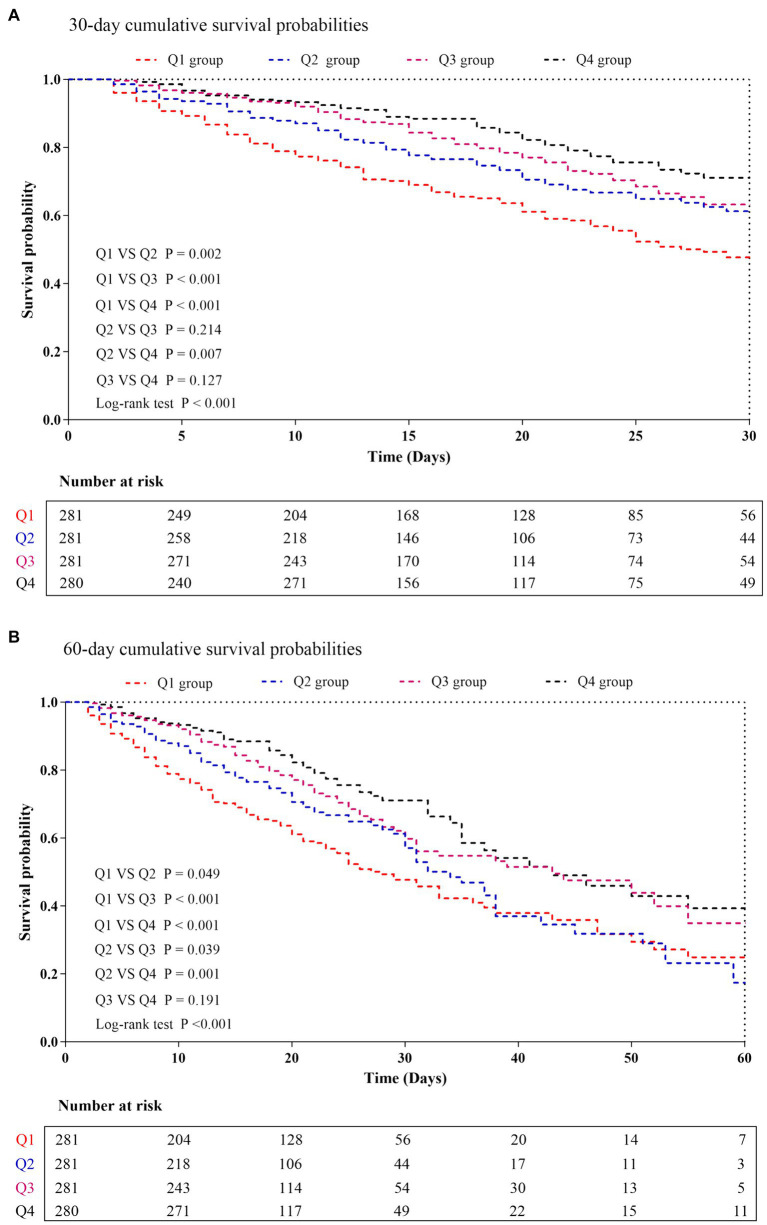
Kaplan–Meier curves showing cumulative probability of all-cause mortality according to groups at 30 days **(A)**, and 60 days **(B)**. ACR quartiles: Q1 group (ACR ≤ 0.169); Q2 group (0.169 < ACR ≤ 0.299); Q3 group (0.299 < ACR ≤ 0.463); Q4 group (ACR > 0.463).

The levels of ACR in the survivor group were found to be significantly higher than those in the non-survivor group (0.335 vs. 0.226, *p* < 0.001). The distribution of ACR, stratified by the mortality status of all-cause in-hospital death, ICU death, and 30-day death, was depicted in Supplementary Figure S1. Kaplan–Meier analysis in Figure 3 illustrated the cumulative incidence of 30−/60-day mortality based on ACR levels. Patients with higher ACR demonstrated a lower risk of 30−/60-day mortality compared to those with lower ACR levels (log-rank *p* < 0.001 for both).

**Figure 4:**
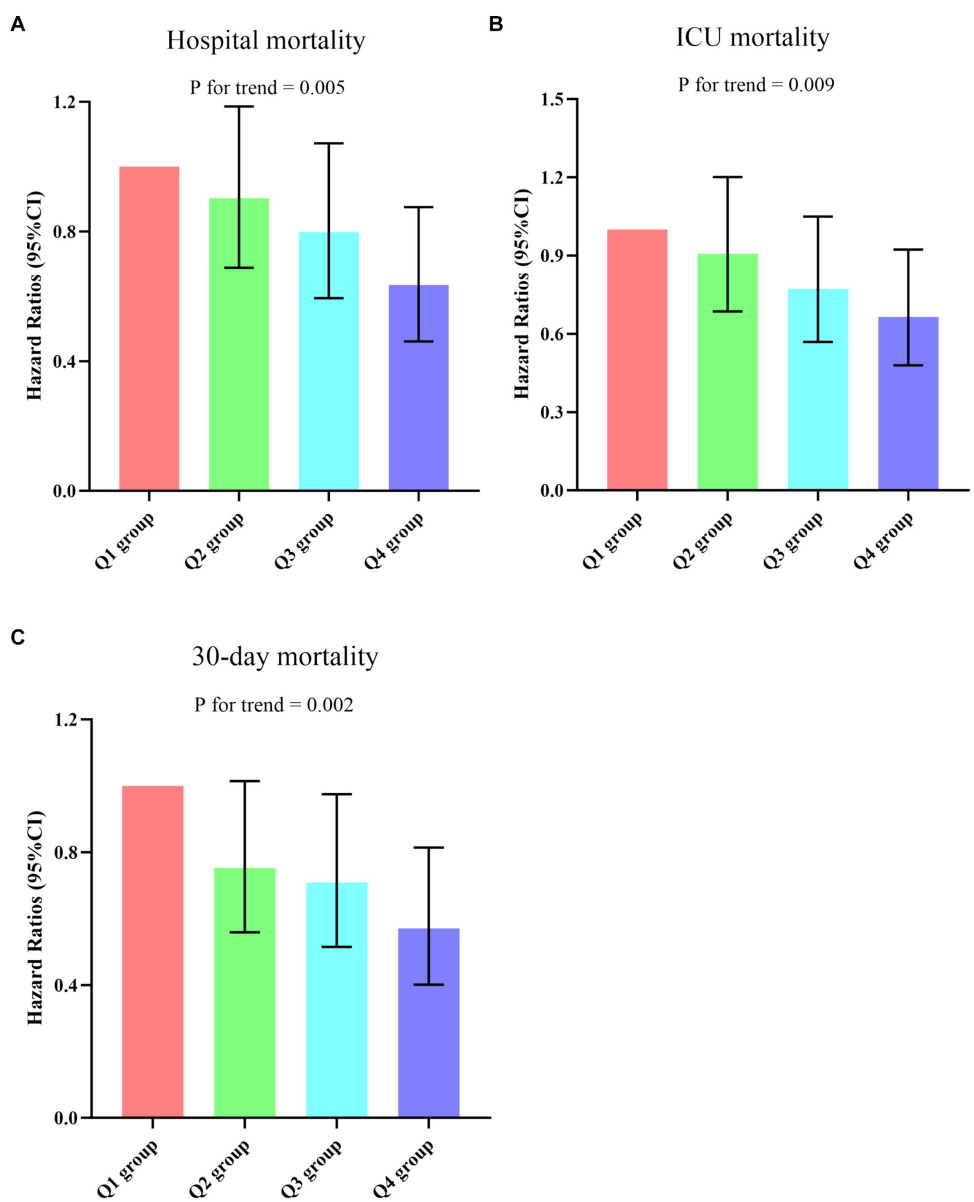
**(A–C)** Hazard ratios (95% CIs) for hospital/ICU/30-day mortality according to ACR quartiles after adjusting for age, gender, BMI, Smoking, hypertension, diabetes, WBC, Neu, Lym, CRP, ALT, AST, glucose, APACHE II score, and SOFA score. Error bars indicate 95% CIs. The first quartile is the reference. ACR quartiles: Q1 group (ACR ≤ 0.169); Q2 group (0.169 < ACR ≤ 0.299); Q3 group (0.299 < ACR ≤ 0.463); Q4 group (ACR > 0.463). ACR, albumin-to-creatinine ratio; BMI, body mass index; WBC, white blood cell; Neu, neutrophil; Lym, lymphocyte; CRP, C-reactive protein; ALT, alanine transaminase; AST, aspartate aminotransferase; APACHE II, Acute Physiology and Chronic Health Evaluation II; SOFA, Sequential Organ Failure Assessment; ICU, Intensive Care Unit.

**Figure 5:**
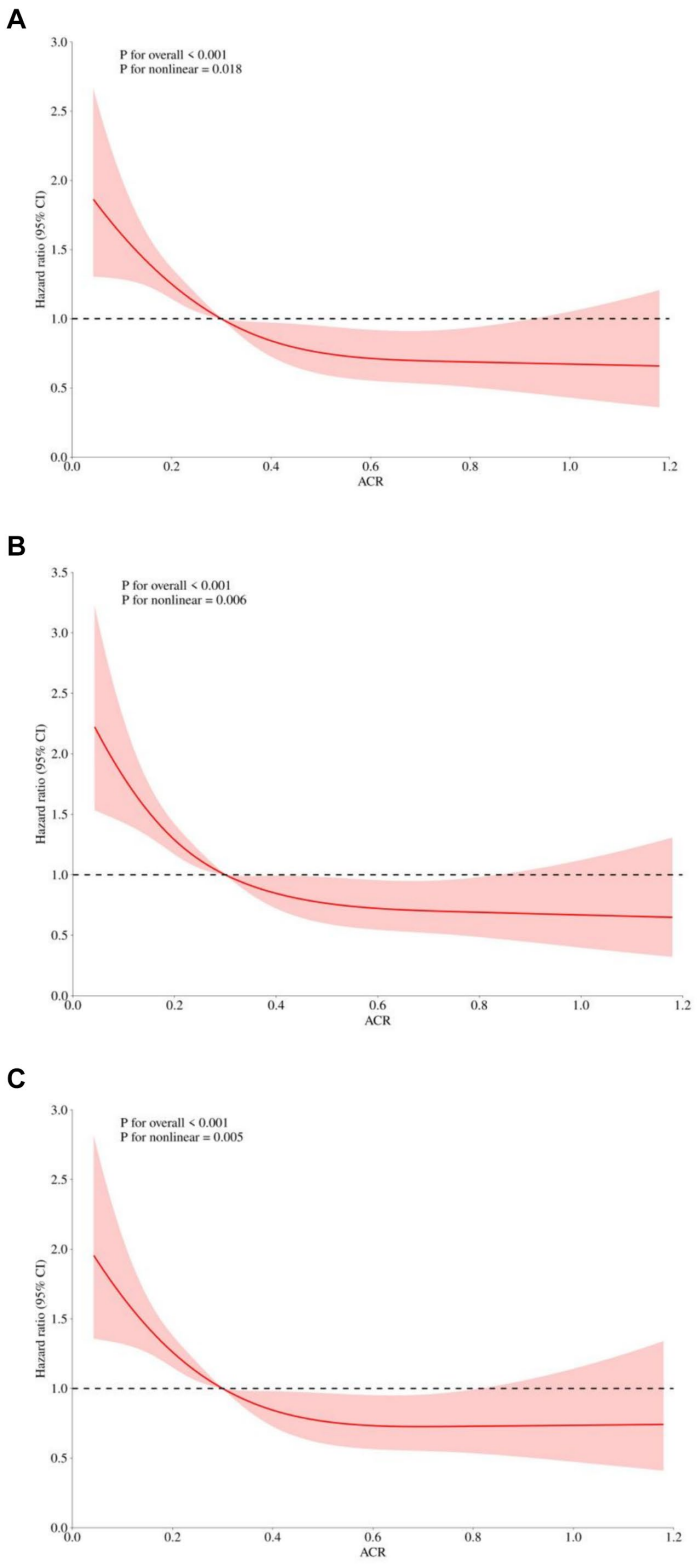
Restricted cubic spline regression analysis of ACR with in hospital all-cause mortality. Heavy central lines represent the estimated adjusted hazard ratios, with shaded ribbons denoting 95% confidence intervals. The horizontal dotted lines represent the hazard ratio of 1.0. **(A)** Restricted cubic spline for hospital mortality. **(B)** Restricted cubic spline for ICU mortality. **(C)** Restricted cubic spline for 30-day mortality. ACR, albumin-to-creatinine ratio; ICU, Intensive Care Unit.

To identify prognostic predictors in patients with sepsis, we performed univariate Cox regression analysis. Variables that showed significance in the univariate analysis (*p* < 0.05) or were potentially associated with clinical outcomes were included as independent variables for Cox regression analysis (Supplementary Table S1). In three multivariable Cox regression analysis models, both the absolute change rate (ACR) increased by 1 unit or 1 SD were significantly and negatively correlated with all-cause in-hospital death, ICU death, and 30-day death (Table 2). When considering ACR as a nominal variable, patients in the higher quartile of ACR were significantly associated with a lower risk of hospital death in the three established Cox proportional hazards models compared to subjects in the lowest quartile. In Model 1, ACR was negatively associated with hospital death [HR (95%CI): 0.474 (0.353–0.638); *p* < 0.001]. After adjusting for confounding factors, the negative association still remained in Model 2 [HR (95%CI): 0.457 (0.339–0.616); *p* < 0.001] and Model 3 [HR (95%CI): 0.635 (0.461–0.875); *p* = 0.005]. The test for trends across tertiles of ACR for the risk of hospital death was statistically significant (Figure 4). Similar results were observed in the multivariate Cox proportional risk analysis of ACR and ICU mortality and 30-day mortality (Table 2). Furthermore, we found a nonlinear negative correlation between these variables, with the HR curve for in-hospital mortality (*p* for non-linear =0.018), ICU mortality (*p* for non-linear =0.005), and 30-day mortality (*p* for non-linear =0.006) first declining sharply as ACR increased and then leveling off (Figure 5).

**Table 2:** Cox proportional hazard ratios (HR) for all-cause mortality.

Variables	Model 1			Model 2			Model 3		
	HR (95% CI)	*p*-value	P for trend	HR (95% CI)	*p*-value	P for trend	HR (95% CI)	*p*-value	P for trend
Hospital mortality									
Per Unit increase	0.271 (0.164–0.448)	<0.001		0.252 (0.151–0.419)	<0.001		0.454 (0.271–0.761)	0.003	
Per SD increase	0.726 (0.642–0.821)	<0.001		0.713 (0.629–0.808)	<0.001		0.824 (0.726–0.935)	0.003	
Quartile^a^			<0.001			<0.001			0.005
Q1 group	Ref			Ref			Ref		
Q2 group	0.787 (0.606–1.021)	0.072		0.758 (0.583–0.986)	0.039		0.903 (0.688–1.186)	0.464	
Q3 group	0.597 (0.454–0.786)	<0.001		0.582 (0.442–0.767)	<0.001		0.798 (0.594–1.072)	0.134	
Q4 group	0.474 (0.353–0.638)	<0.001		0.457 (0.339–0.616)	<0.001		0.635 (0.461–0.875)	0.005	
ICU mortality									
Per Unit increase	0.283 (0.169–0.474)	<0.001		0.265 (0.157–0.447)	<0.001		0.498 (0.293–0.847)	0.010	
Per SD increase	0.734 (0.647–0.833)	<0.001		0.722 (0.635–0.821)	<0.001		0.843 (0.740–0.960)	0.010	
Quartile^a^			<0.001			<0.001			0.009
Q1 group	Ref			Ref			Ref		
Q2 group	0.781 (0.597–1.022)	0.071		0.755 (0.576–0.989)	0.041		0.907 (0.686–1.201)	0.496	
Q3 group	0.573 (0.431–0.762)	<0.001		0.557 (0.419–0.742)	<0.001		0.772 (0.569–1.049)	0.098	
Q4 group	0.486 (0.359–0.657)	<0.001		0.471 (0.347–0.638)	<0.001		0.665 (0.479–0.923)	0.015	
30-day mortality									
Per Unit increase	0.208 (0.116–0.373)	<0.001		0.198 (0.110–0.358)	<0.001		0.399 (0.218–0.730)	0.003	
Per SD increase	0.680 (0.589–0.785)	<0.001		0.673 (0.582–0.778)	<0.001		0.798 (0.689–0.926)	0.003	
Quartile^a^			<0.001			<0.001			0.002
Q1 group	Ref			Ref			Ref		
Q2 group	0.655 (0.492–0.872)	0.004		0.637 (0.477–0.850)	0.002		0.753 (0.559–1.014)	0.062	
Q3 group	0.536 (0.398–0.721)	<0.001		0.529 (0.393–0.713)	<0.001		0.709 (0.515–0.975)	0.035	
Q4 group	0.406 (0.292–0.564)	<0.001		0.396 (0.285–0.552)	<0.001		0.571 (0.401–0.814)	0.002	

a ACR: Q1 group (ACR ≤ 0.169); Q2 group (0.169 < ACR ≤ 0.299); Q3 group (0.299 < ACR ≤ 0.463); Q4 group (ACR > 0.463).

Model 1: unadjusted.

Model 2: adjusted for age, gender, BMI, Smoking, hypertension, and diabetes.

Model 3: adjusted for age, gender, BMI, Smoking, hypertension, diabetes, WBC, Neu, Lym, CRP, ALT, AST, glucose, APACHE II score, and SOFA score.

ACR, albumin-to-creatinine ratio; BMI, body mass index; WBC, white blood cell; Neu, neutrophil; Lym, lymphocyte; CRP, C-reactive protein; ALT, alanine transaminase; AST, aspartate aminotransferase; APACHE II, Acute Physiology and Chronic Health Evaluation II; SOFA, Sequential Organ Failure Assessment; ICU, Intensive Care Unit.

### Subgroup analysis

To further explore whether ACR remained an independent prognostic factor in specific subgroups of patients with sepsis, we conducted an exploratory subgroup analysis based on age (≤65 or > 65), gender (male or female), BMI (≤22.49 or > 22.49), smoking (yes or no), hypertension (yes or no), diabetes (yes or no), and SOFA score (≤12 or > 12). The associations between ACR and hospital mortality, ICU mortality, and 30-day mortality were generally consistent across the subgroups (Tables 3–5). We did not observe a significant interaction between ACR and age, gender, BMI, smoking, hypertension, diabetes, or SOFA score (all *p*-values for interaction >0.05). This indicated that ACR was an independent prognostic factor.

**Table 3:** Subgroup analysis regarding the influence of different ACR in the hospital mortality.

Subgroups	No. hospital mortality/No. patients	HR (95% CI)	*p*-value	P for interaction
Age				0.836
>65	315/840	0.281 (0.162–0.490)	<0.001	
≤65	64/283	0.233 (0.068–0.798)	0.020	
Gender				0.604
Male	251/707	0.236 (0.122–0.454)	<0.001	
Female	128/416	0.317 (0.143–0.704)	0.005	
BMI				0.833
>22.49	150/549	0.284 (0.128–0.628)	0.002	
≤22.49	229/574	0.257 (0.134–0.493)	<0.001	
Smoking				0.428
Yes	88/230	0.175 (0.055–0.559)	0.003	
No	291/893	0.300 (0.172–0.523)	<0.001	
Hypertension				0.760
Yes	211/579	0.237 (0.122–0.462)	<0.001	
No	168/544	0.286 (0.131–0.625)	0.002	
Diabetes				0.093
Yes	111/309	0.135 (0.049–0.370)	<0.001	
No	268/814	0.363 (0.203–0.651)	0.001	
SOFA				0.822
>12	221/488	0.355 (0.189–0.667)	0.001	
≤12	158/635	0.301 (0.137–0.662)	0.003	

ACR, albumin-to-creatinine ratio; BMI, body mass index; SOFA, Sequential Organ Failure Assessment.

**Table 4:** Subgroup analysis regarding the influence of different ACR in the ICU mortality.

Subgroups	No. ICU mortality/No. patients	HR (95% CI)	*p*-value	P for interaction
Age				0.802
>65	297/840	0.294 (0.166–0.519)	<0.001	
≤65	61/283	0.237 (0.068–0.830)	0.024	
Gender				
Male	238/707	0.231 (0.117–0.454)	<0.001	0.397
Female	120/416	0.367 (0.164–0.821)	0.015	
BMI				
>22.49	142/549	0.277 (0.122–0.630)	0.002	0.969
≤22.49	216/574	0.280 (0.144–0.544)	<0.001	
Smoking				
Yes	85/230	0.160 (0.049–0.524)	0.002	0.293
No	273/893	0.324 (0.183–0.572)	<0.001	
Hypertension				
Yes	199/579	0.248 (0.125–0.491)	<0.001	0.787
No	159/544	0.299 (0.135–0.664)	0.003	
Diabetes				
Yes	104/309	0.121 (0.042–0.352)	<0.001	0.051
No	254/814	0.398 (0.220–0.719)	0.002	
SOFA				
>12	210/488	0.368 (0.193–0.701)	0.002	0.843
≤12	148/635	0.324 (0.144–0.728)	0.006	

ACR, albumin-to-creatinine ratio; BMI, body mass index; SOFA, Sequential Organ Failure Assessment; ICU, Intensive Care Unit.

**Table 5:** Subgroup analysis regarding the influence of different ACR in the 30-day mortality.

Subgroups	No. ICU mortality/No. patients	HR (95% CI)	*p*-value	P for interaction
Age				0.448
>65	266/840	0.236 (0.125–0.446)	<0.001	
≤65	50/283	0.121 (0.027–0.541)	0.006	
Gender				0.290
Male	202/707	0.155 (0.070–0.341)	<0.001	
Female	114/416	0.289 (0.121–0.690)	0.005	
BMI				0.712
>22.49	124/549	0.239 (0.095–0.601)	0.002	
≤22.49	192/574	0.188 (0.088–0.400)	<0.001	
Smoking				0.701
Yes	78/230	0.165 (0.049–0.556)	0.004	
No	238/893	0.220 (0.113–0.428)	<0.001	
Hypertension				0.603
Yes	173/579	0.237 (0.110–0.511)	<0.001	
No	143/544	0.176 (0.071–0.436)	<0.001	
Diabetes				0.403
Yes	90/309	0.137 (0.044–0.431)	0.001	
No	226/814	0.246 (0.124–0.487)	<0.001	
SOFA				0.524
>12	188/488	0.314 (0.153–0.644)	0.002	
≤12	128/635	0.219 (0.087–0.553)	0.001	

ACR, albumin-to-creatinine ratio; BMI, body mass index; SOFA, Sequential Organ Failure Assessment.

## Discussion

This study represented the first investigation into the correlation between ACR and clinical outcomes in critically ill patients with sepsis. The key findings presented suggested that a decrease in ACR may serve as an independent risk factor contributing to in-hospital mortality among sepsis patients. This conclusion was consistent with previous observations of ICU and 30-day mortality rates in sepsis patients, demonstrating a reliable association. Importantly, this relationship remained significant even after adjusting for various clinical and laboratory variables. Additionally, the RCS curve analysis revealed a negative and non-linear correlation between ACR levels and the risk of in-hospital, ICU, and 30-day mortality. Given its cost-effectiveness and widespread availability in clinical settings, ACR had the potential to serve as a valuable prognostic indicator for critically ill patients with sepsis.

The precise mechanisms underlying the relationship between ACR and the risk of adverse outcomes in sepsis patients had yet to be fully elucidated. However, it was known that inflammation and oxidative stress played significant roles in the onset and progression of sepsis (11, 12). Alb, a commonly measured parameter in clinical laboratories, had a profound impact on various physiological processes (13). Previous studies had indicated that decreased synthesis and increased breakdown of serum Alb were associated with inflammation (25). Inflammatory states can lead to increased microvascular permeability, altering the distribution of Alb between the intra- and extravascular compartments and resulting in reduced serum Alb levels in critically ill patients (26). Arnau-Barrés et al. (27) had shown that serum Alb level < 2.6 g/dL can be used as a prognosis factor for 30-day mortality among elderly patients with sepsis. Cao et al. (28) demonstrated that Alb level was associated with 28-day, 60-day, 180-day and 1-year mortality in sepsis patients. Kumar et al. (29) also confirmed that hypoalbuminemia may serve as an indicator of severity and adverse prognosis in sepsis. Furthermore, decreased serum Alb had been linked to the promotion of oxidative stress, further increasing the risk of mortality in sepsis patients (30, 31).

Early onset of multiple organ damage was a well-known predictor of in-hospital and long-term mortality in sepsis patients, with acute kidney injury (AKI) being a common occurrence (32, 33). Sepsis-induced AKI was associated with an elevated risk of subsequent chronic kidney disease and was linked to increased morbidity and mortality rates in sepsis cases (34, 35). Elevated levels of serum creatinine, an indicator of acute renal impairment, played a crucial role in assessing oxidative stress and inflammatory status, all of which directly impacted the prognosis of sepsis (36). Research conducted by Xiao et al. (20) demonstrated that increased serum creatinine levels were prevalent among sepsis patients and were associated with 28-day mortality. Additionally, these elevated levels served as an independent risk factor for higher mortality rates. Vanmassenhove et al. (37) suggested that changes in serum creatinine within the first 24 h of admission were associated with mortality, with an increase of 0.3 mg/mL in creatinine demonstrating predictive value for sepsis-related death. Therefore, serum creatinine, as a biomarker of AKI, had the potential to reflect the severity of sepsis and aided in the identification of high-risk patients.

Currently, the diagnosis of sepsis using a combination of multiple biochemical markers was a highly researched topic (38–40). While the association between serum Alb and creatinine levels and sepsis had been supported by previous scientific evidence, there had been controversies regarding the factors that influenced their concentrations, such as nutritional status, chronic inflammation, and chronic kidney disease (17). Taking these factors into consideration, we propose using the ratio of serum Alb to serum creatinine to assess the relationship between ACR and clinical outcomes in sepsis. ACR was a reproducible and cost-effective parameter that can be easily collected during routine clinical management and had been frequently used to reflect the body’s inflammatory state and oxidative stress (21). Therefore, this investigation enriches the recent evidence that ACR level can be used to predict renal outcomes in type 2 diabetic and prediabetic subjects (41). Liu et al. (42) demonstrated that ACR can serve as a useful biomarker for predicting all-cause mortality and adverse outcomes in a cohort of 2,250 patients with early-phase acute myocardial infarction (AMI) in the emergency department (ED). Shen et al. (22) discovered that preoperative ACR levels were significant independent predictors of postoperative respiratory complications, renal complications, liver injury, infections, and in-hospital death in patients undergoing heart transplantation. Additionally, a study by Turkyilmaz et al. (43) revealed that ACR was an independent predictor of in-hospital mortality, contrast-induced nephropathy, congestive heart failure, and stent thrombosis at 30 days among patients with ST-segment elevation myocardial infarction (STEMI). However, no research had been conducted to investigate the association between ACR and the risk of poor outcomes in patients with sepsis. Therefore, our study represented the first evaluation of the predictive potential of ACR for all-cause mortality in sepsis patients, and confirmed that ACR was a promising indicator that independently predicted in-hospital, ICU, and 30-day mortality in sepsis patients. We also explored the joint association of ACR with in-hospital, ICU, and 30-day mortality among different subgroups. The results remained consistent in sensitivity analysis and subgroup analysis, indicating that the predictive value of ACR for all-cause mortality was applicable to almost all populations. In summary, these findings suggested that ACR was a readily available and objective hematological biomarker for systemic inflammation.

Apart from inflammation, several other potential mechanisms may contribute to the pathophysiology of sepsis: (1) Sepsis stimulated endothelial cells to produce nitric oxide, which led to vasodilation and a loss of autoregulation, resulting in endothelial dysfunction. This dysfunction can easily trigger microthrombus formation and capillary obstruction, leading to insufficient microcirculatory perfusion in the local renal tissue. Consequently, this prolonged the exposure of renal tubular epithelial cells to inflammatory mediators, leading to cellular damage (44). Existing studies had confirmed that biomarkers of endothelial dysfunction, such as the microalbuminuria to creatinine ratio and adrenomedullin, can serve as indicators for predicting the prognosis of septic patients (45–47). (2) Patients with sepsis were in a state of high catabolism and hypermetabolism, which resulted in the excessive consumption of energy. This increased the metabolism and breakdown of fats and proteins, subsequently leading to a decrease in Alb levels (48, 49). The ACR leveraged early laboratory observations that creatinine levels were positively associated with sepsis, while albumin levels were negatively associated. This ratio enhanced the predictive potential for sepsis.

However, there were some limitations to this study that should be taken into consideration. Firstly, our study was a retrospective study conducted at a single center, which limited our ability to establish a causal relationship between ACR and sepsis compared to prospective studies. Consequently, our findings may lack some persuasive power. Secondly, while repeated measurements of serum Alb and creatinine levels during hospitalization can enhance risk assessment in sepsis patients, we only measured ACR once upon admission. Therefore, the potential variations in ACR over the course of hospitalization and its predictive value for adverse events were not explored in this study. Thirdly, the sample size in our study was relatively small, and the follow-up period was relatively short, particularly during subgroup analysis. Lastly, since our study population consisted solely of adults from China, it was necessary to conduct further research to determine whether the association between ACR and poor outcomes extended to other demographic groups, including minors and individuals from different countries. In future studies, we intend to conduct more comprehensive laboratory investigations with larger participant cohorts and consider additional confounding factors to delve deeper into the underlying mechanisms.

## Conclusion

In summary, our study revealed that a low ACR was correlated with a higher risk of all-cause mortality in sepsis patients. Assessing the ACR could serve as a useful tool for stratifying individuals with sepsis at a heightened risk of unfavorable outcomes. This valuable information can aid healthcare professionals in implementing timely early interventions and developing effective clinical plans to improve patient outcomes.

## Data Availability

The raw data supporting the conclusions of this article will be made available by the authors, without undue reservation.
