# Supplementary material for: Elevated serum albumin-to-creatinine ratio as a protective factor on clinical outcomes among critically ill patients with sepsis: a retrospective study
Source: Front Med (Lausanne). 2024 Sep 19;11:1436533. doi: 10.3389/fmed.2024.1436533 (PMC11446770; doi:10.3389/fmed.2024.1436533)
Supplement: Supplementary file 1 [file Table_1.DOCX]

**Supplementary material**

Supplementary Table 1: Cox proportional hazards regression of the factors influencing all-cause death of the study population.

Supplementary Figure 1: Boxplots of the ACR showing the distribution in the Survivor group and Non-survivor group.

Table S1. Cox proportional hazards regression of the factors influencing all cause mortality of the study population.

| **Variables** | **HR** | **95% CI** | **P-value** |
| --- | --- | --- | --- |
| ACR | 0.271 | 0.164-0.448 | <0.001 |
| Age | 1.019 | 1.011-1.027 | <0.001 |
| Male | 0.953 | 0.770-1.180 | 0.660 |
| BMI | 0.976 | 0.953-1.000 | 0.048 |
| Smoking | 1.145 | 0.901-1.456 | 0.269 |
| Hypertension | 1.161 | 0.948-1.423 | 0.149 |
| Diabetes | 1.153 | 0.924-1.439 | 0.206 |
| WBC | 1.014 | 1.001-1.026 | 0.028 |
| Neu | 1.019 | 1.005-1.032 | 0.005 |
| Lym | 0.489 | 0.378-0.631 | <0.001 |
| CRP | 1.002 | 1.000-1.003 | 0.006 |
| ALT | 1.009 | 0.992-1.025 | 0.299 |
| AST | 1.001 | 1.000-1.001 | <0.001 |
| Glucose | 1.000 | 1.000-1.000 | <0.001 |
| APACHE II score | 1.044 | 1.032-1.057 | <0.001 |
| SOFA score | 1.078 | 1.052-1.105 | <0.001 |

Abbreviations: ACR, albumin-to-creatinine ratio; BMI, body mass index; WBC, white blood cell; Neu, neutrophil; Lym, lymphocyte; CRP, C-reactive protein; ALT, alanine transaminase; AST, aspartate aminotransferase; APACHE II, Acute Physiology and Chronic Health Evaluation II; SOFA, Sequential Organ Failure Assessment.


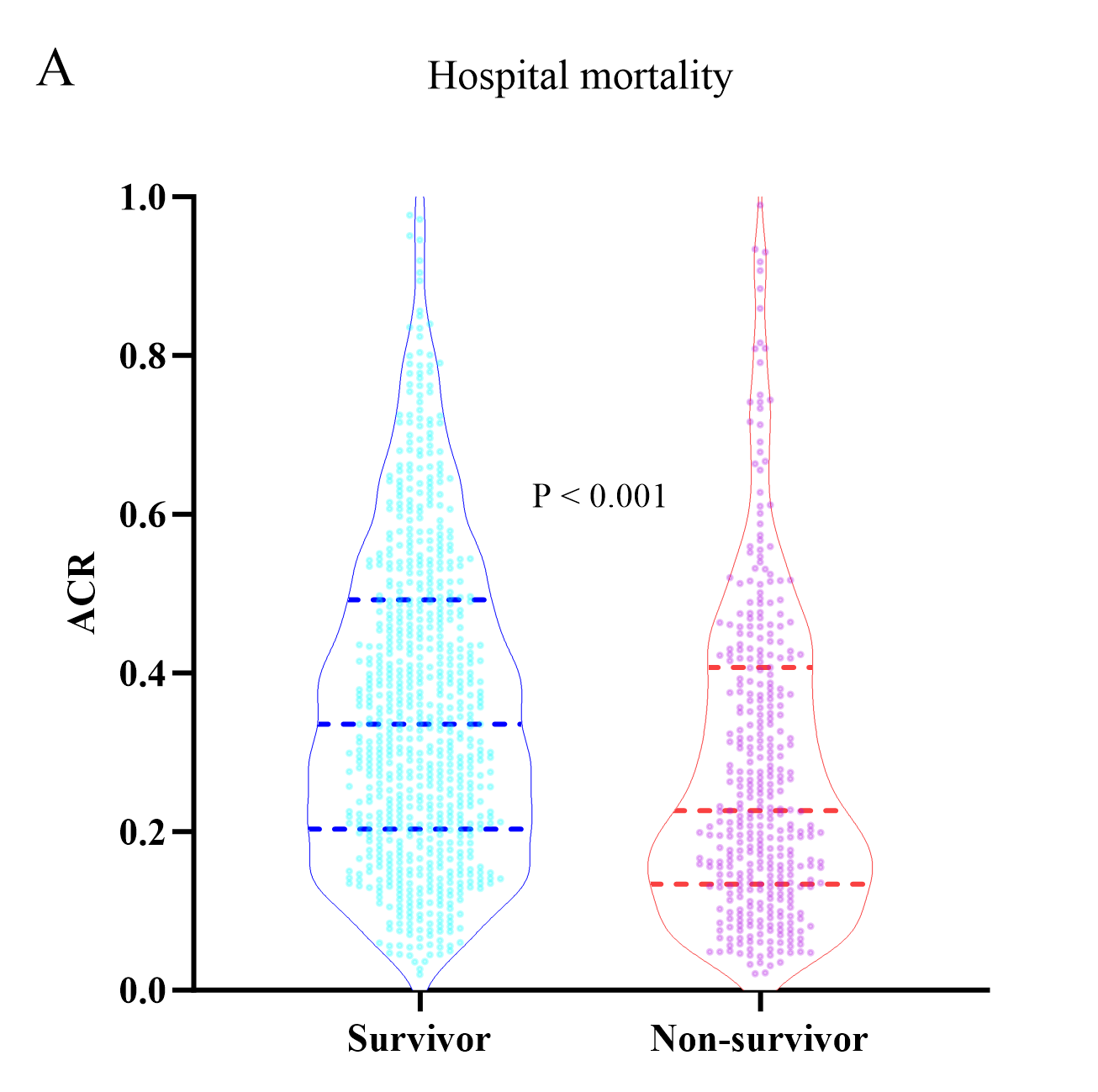

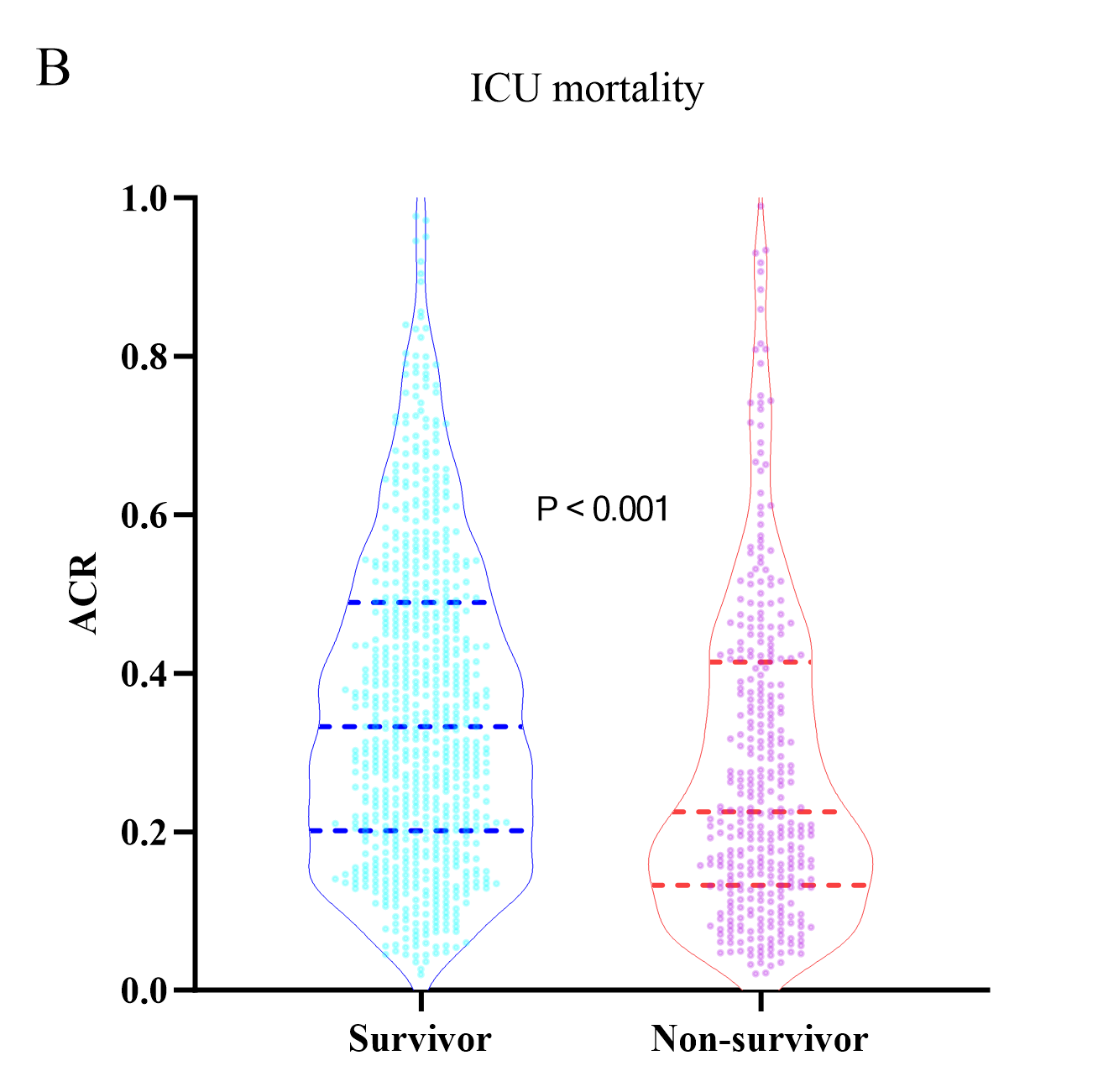

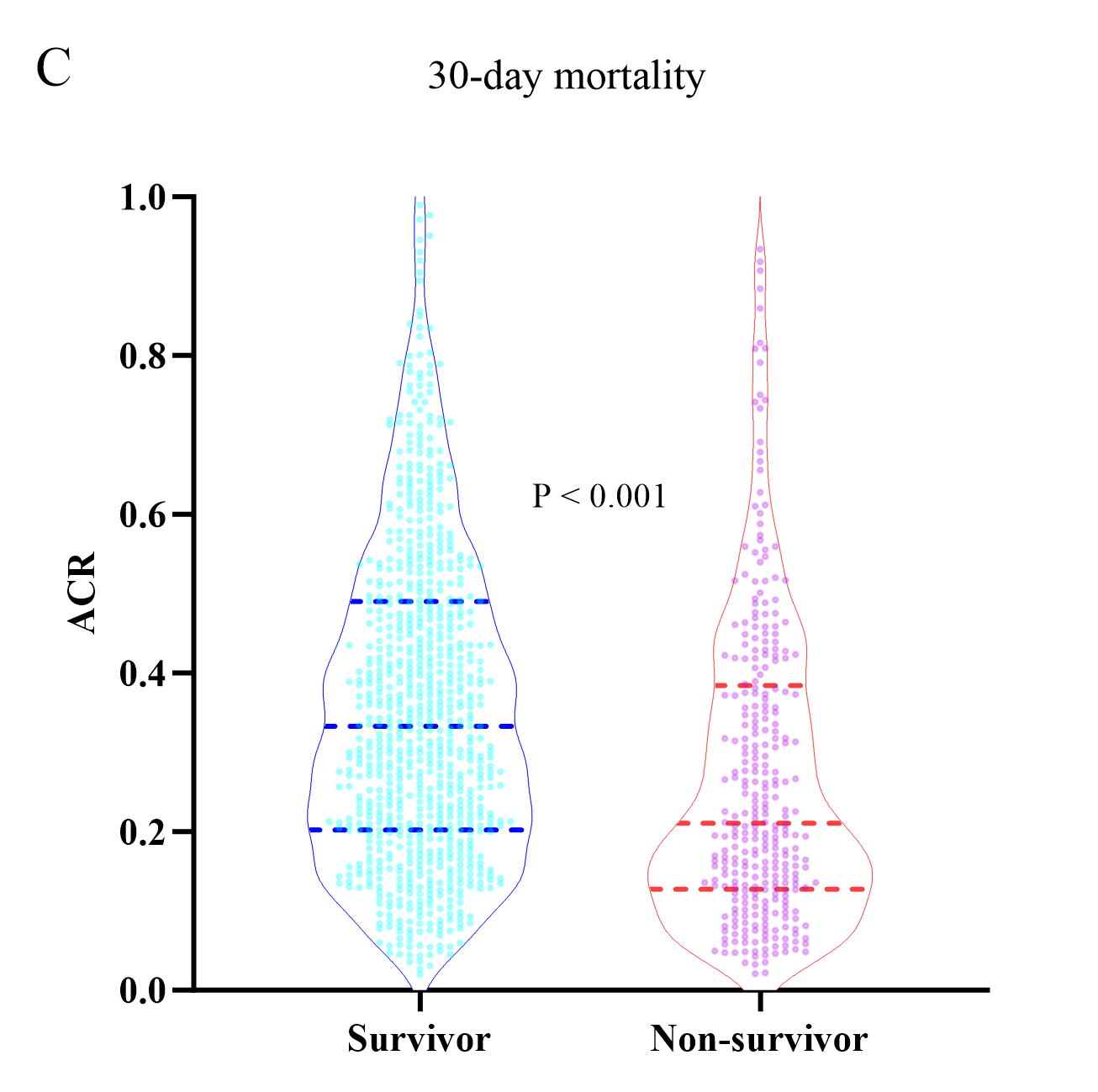


Figure S1. A. Boxplots of the ACR showing the distribution in the Survivor group and Non-survivor group during hospital stay. B. Boxplots of the ACR showing the distribution in the Survivor group and Non-survivor group during ICU stay. B. Boxplots of the ACR showing the distribution in the Survivor group and Non-survivor group within 30 days after admission to the ICU. Abbreviations: ACR, albumin-to-creatinine ratio; ICU, Intensive Care Unit.
